# Implementation of Fully Automated AI-Integrated System for Body Composition Assessment on Computed Tomography for Opportunistic Sarcopenia Screening: Multicenter Prospective Study

**DOI:** 10.2196/69940

**Published:** 2025-09-05

**Authors:** Bushra Urooj, Yousun Ko, Seongwon Na, In-One Kim, Eun-Hee Lee, Seon Cho, Heeryeol Jeong, Seungwoo Khang, Jeongjin Lee, Kyung Won Kim

**Affiliations:** 1 Department of Medical Science, Asan Medical Institute of Convergence Science and Technology, University of Ulsan College of Medicine, 88 Olympic-ro 43-gil, Asan Medical Center, Seoul, 05505, Republic of Korea; 2 Department of Radiology, Asan Medical Center, University of Ulsan College of Medicine, 88 Olympic-ro 43-gil, Asan Medical Center, Seoul, 05505, Republic of Korea; 3 Medicheck Research Institute, Korea Association of Health Promotion, Seoul, Republic of Korea; 4 School of Computer Science and Engineering, Soongsil University, Seoul, Republic of Korea

**Keywords:** artificial intelligence, body composition, sarcopenia, myosteatosis, opportunistic screening, health check-up

## Abstract

**Background:**

Opportunistic computed tomography (CT) screening for the evaluation of sarcopenia and myosteatosis has been gaining emphasis. A fully automated artificial intelligence (AI)–integrated system for body composition assessment on CT scans is a prerequisite for effective opportunistic screening. However, no study has evaluated the implementation of fully automated AI systems for opportunistic screening in real-world clinical practice for routine health check-ups.

**Objective:**

The aim of this study is to evaluate the performance and clinical utility of a fully automated AI-integrated system for body composition assessment on opportunistic CT during routine health check-ups.

**Methods:**

This prospective multicenter study included 537 patients who underwent routine health check-ups across 3 institutions. Our AI algorithm models are composed of selecting L3 slice and segmenting muscle and fat area in an end-to-end manner. The AI models were integrated into the Picture Archiving and Communication System (PACS) at each institution. Technical success rate, processing time, and segmentation accuracy in Dice similarity coefficient were assessed. Body composition metrics were analyzed across age and sex groups.

**Results:**

The fully automated AI-integrated system successfully retrieved anonymized CT images from the PACS, performed L3 selection and segmentation, and provided body composition metrics, including muscle quality maps and muscle age. The technical success rate was 100% without any failed cases requiring manual adjustment. The mean processing time from CT acquisition to report generation was 4.12 seconds. Segmentation accuracy comparing AI results and human expert results was 97.4%. Significant age-related declines in skeletal muscle area and normal-attenuation muscle area were observed, alongside increases in low-attenuation muscle area and intramuscular adipose tissue.

**Conclusions:**

Implementation of the fully automated AI-integrated system significantly enhanced opportunistic sarcopenia screening, achieving excellent technical success and high segmentation accuracy without manual intervention. This system has the potential to transform routine health check-ups by providing rapid and accurate assessments of body composition.

## Introduction

Sarcopenia, characterized by the progressive loss of skeletal muscle mass and function, and myosteatosis, defined as excessive fat accumulation within muscle tissue, are critical health issues associated with functional impairment, frailty, and increased mortality [1]. With the global population aging, the prevalence of these conditions is expected to rise significantly [2]. Early diagnosis is essential to mitigate these risks by enabling timely management [3]. Thus, routine health screening for sarcopenia and myosteatosis is important for preventive health care.

The imaging diagnosis of sarcopenia relies on assessing both muscle mass and quality. While magnetic resonance imaging and whole-body dual-energy X-ray absorptiometry provide accurate evaluations, their limited accessibility and longer imaging times pose significant barriers to widespread clinical adoption [3]. In contrast, computed tomography (CT), widely used for routine diagnostic purposes, has emerged as a practical imaging modality for body composition assessment [4].

Especially, opportunistic CT screening, where CT scans performed for other clinical indications are repurposed for secondary analyses, represents an innovative approach to diagnosing sarcopenia without additional costs or radiation exposure [5]. The development of automated body composition assessment methods powered by artificial intelligence (AI) has further streamlined this process, making opportunistic CT screening a practical option in daily clinical practice [6]. Integration of such AI solutions into clinical workflows, particularly through Picture Archiving and Communication Systems (PACS), is essential for establishing standardized processes for fully automated opportunistic CT screening.

Although some studies have explored opportunistic CT screening for sarcopenia [7,8], no study has prospectively evaluated the seamless workflow integration, technical success, and processing efficiency of fully AI-integrated systems in routine health check-up settings. Therefore, this study aims to implement and evaluate the fully automated AI-integrated opportunistic CT screening system for routine health check-ups. By assessing its technical success, processing efficiency, and diagnostic outcomes across multiple health check-up institutions, this study seeks to demonstrate the system’s scalability, robustness, and clinical utility.

## Methods

### Recruitment

Designed as a multicenter prospective observational study, it included patients who underwent health check-ups at 3 health institutions affiliated with the Korea Association of Health Promotion (KAHP) between December 2022 and December 2023. Consecutive recruitment was implemented according to inclusion and exclusion criteria. Inclusion criteria were adults aged 20 years or older who had undergone abdominal CT scans as part of their routine health check-ups. Exclusion criteria were patients with canceled or incomplete CT scans, or those with significant artifacts on CT images. No specific screening for comorbidities such as diabetes or prior malignancies was performed. We included all patients who underwent abdominal CT, regardless of contrast enhancement.

### CT Acquisition

CT scans were performed using Revolution Discovery (GE Healthcare), Somatom Definition AS+, and Somatom Definition Edge (Siemens Healthineers) scanners, using parameters as follows: tube voltage, 120 kVp; effective tube current, 200 reference mAs (Care Dose 4D; Siemens Healthineers) or 100‐400 mA (AutomA or SmartmA; GE Healthcare); field of view, 30‐40 cm; collimation, 0.31‐0.75 mm; and pitch, 0.98‐1.00. Images were reconstructed using the filtered backprojection technique with the soft tissue reconstruction algorithm at a section thickness of 5 mm with no interslice gap. To ensure that variations between scanners did not affect segmentation accuracy or Hounsfield Unit (HU) measurements, we standardized acquisition protocols with the same kVp, reconstruction technique, and slice thickness.

### AI Algorithm Model

**Figure 1.**
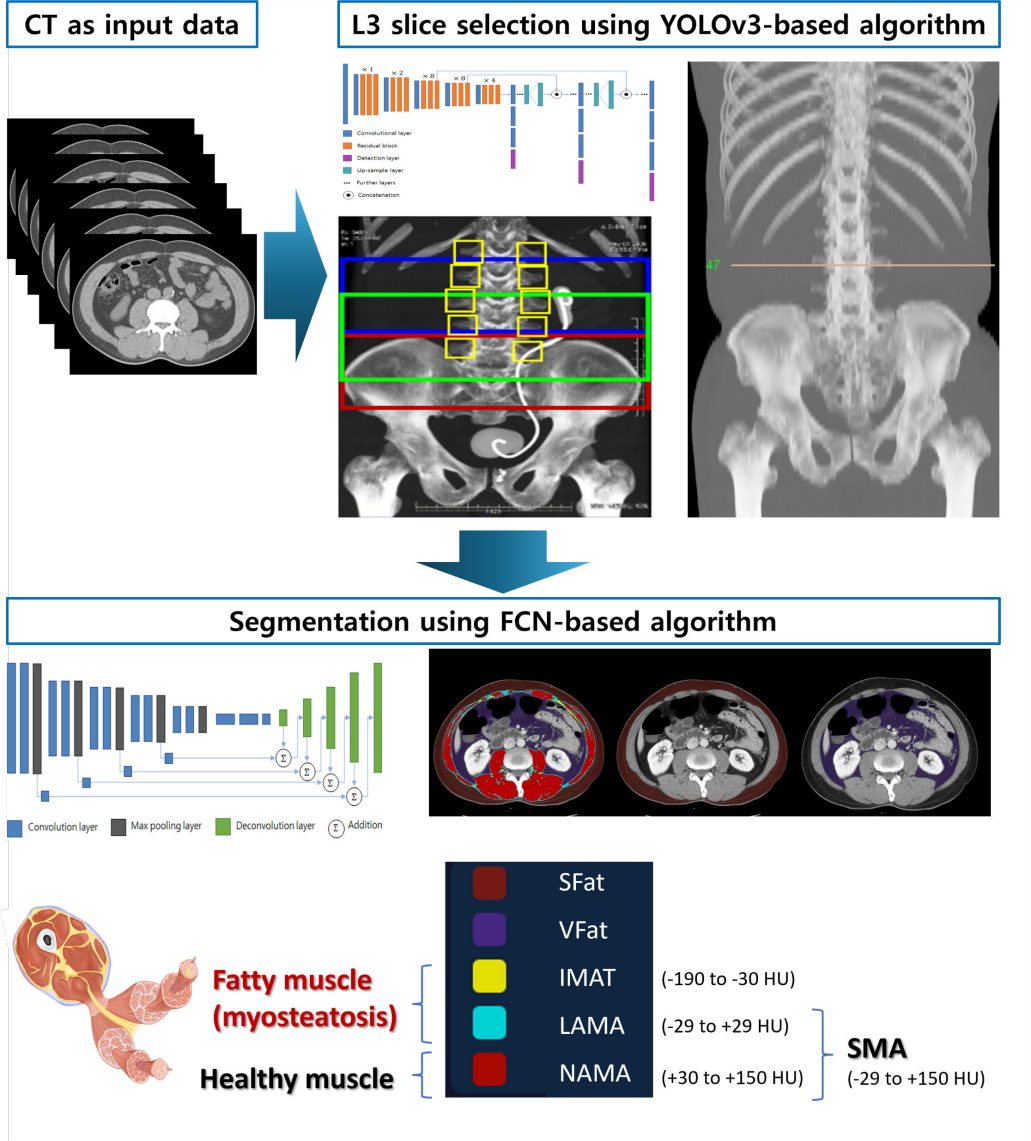
AI algorithm models. CT: computed tomography; FCN: fully convolution network; IMAT: inter/intramuscular adipose tissue; LAMA: low-attenuation muscle area; NAMA: normal-attenuation muscle area; SFAT: subcutaneous fat area; SMA: skeletal muscle area; VFAT: visceral fat area; YOLOv3: You Only Look Once v3.

The AI solution (Aid-U, iAID Inc.), a fully automatic deep learning solution for both L3 selection and body composition assessment, was used for the AI algorithm model (Video S1 in Multimedia Appendix 1). The software uses a You Only Look Once v3 (YOLOv3)–based algorithm for automatic L3 inferior endplate level selection and a fully convolution network for segmentation of abdominal muscle area and fat areas, as illustrated in Figure 1 [9]. Selected L3 level CT images were automatically segmented to generate the boundary of total abdominal muscles and measured the abdominal muscle and fat area [10]. Based on predefined HU thresholds, our system then classified the segmented muscle into normal-attenuation muscle area (NAMA, +30 HU to +150 HU), low-attenuation muscle area (LAMA, −29 HU to +29 HU), and inter- and intramuscular adipose tissue (IMAT, −190 HU to −30 HU). The distinction between NAMA, LAMA, and IMAT was made to differentiate healthy muscle from fatty infiltration and to assess myosteatosis. This approach allows a more detailed assessment of muscle quality [11,12]. We defined a skeletal muscle area (SMA) by combining NAMA and LAMA. The BMI-adjusted SMA is defined by SMA/BMI. The visceral fat area (VFAT) and the subcutaneous fat area (SFAT) were demarcated using fat tissue thresholds of −190 HU to −30 HU information.

### Setup for Fully Automated AI-Integrated System

**Figure 2.**
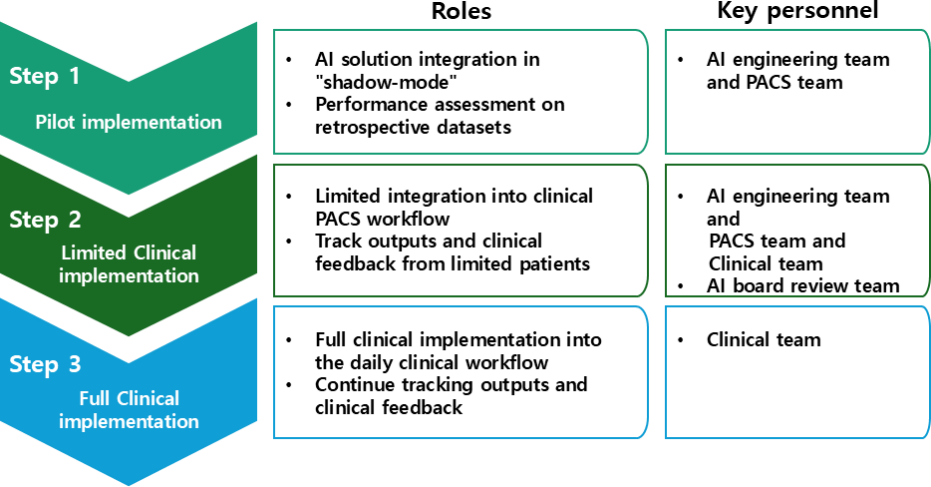
Step-by-step AI implementation and integration in clinical practice. AI: artificial intelligence; PACS: Picture Archiving and Communication System.

To ensure seamless compatibility with existing clinical workflows and institutional imaging infrastructures, the AI model (Aid-UTM, iAID Inc.) was directly integrated with the PACS (INFINITT PACS, Infinitt Co.) at the participating health institutions. Processes for the fully automated AI-integrated opportunistic CT screening system were established in a step-by-step manner, following the most recent implementation guidelines, as illustrated in Figure 2 [13].

The first step was pilot implementation. A project kick-off meeting was conducted with the AI engineering team, the clinical team from KABP, and the PACS team to ensure seamless coordination. During this phase, the AI solution servers were integrated with the PACS infrastructure. Retrospective testing was performed using test CT datasets (n=10) obtained from the open data repository of the 2022 Body Morphometry AI Segmentation Challenge. The second step involved limited clinical deployment. CT scans from the first 10 patients enrolled in this study were used to evaluate real-time AI processing in a prospective manner. An internal monitoring tool was deployed alongside this step to track CT data transfer and AI processing performance, ensuring robustness and transparency. The final step was full clinical deployment, enabling the system for long-term use in routine health check-up settings. This phase marked the integration of the AI system into daily clinical workflows for opportunistic CT screening, ensuring scalability and sustained performance.

### Statistical Analysis

The technical success rate of the AI system was calculated as the proportion of CT scans successfully processed without requiring manual adjustments. The overall processing time was reported as the mean time interval from the completion of CT scan acquisition to the availability of the AI-generated report. Segmentation accuracy was assessed by comparing AI-generated results with manual annotations by radiologists, with agreement quantified using the Dice similarity coefficient (DSC).

To evaluate age-related and gender-specific differences in body composition metrics, 1-way ANOVA was conducted across age groups. *P*<.05 was considered statistically significant.

Our study proposed the concept of muscle age, defined as the estimated age at which the participant’s muscle mass corresponds to the average muscle mass of that age group. Muscle age is calculated using data from Kim et al’s study [14], which includes 11,845 healthy Korean adults who underwent abdominal CT scans during routine health check-ups. Participants with known malignancy, chronic diseases, or metabolic disorders were excluded. SMA and SMA/BMI values were stratified by sex and age. Participants aged 35 to 65 years, representing the 5th to 95th percentile of the healthy adult population, were used to generate sex-specific regression models for estimating muscle age. First, we regressed SMA/BMI on a constant and age in a sample of participants aged between 35 and 65 years, representing approximately the 5th and 95th percentiles for age in both sexes. Next, we derived the inverse of the regression equation by solving for age as a function of SMA/BMI. The SE of the estimated age was computed using the delta method, based on the original variance-covariance matrix adjusted for heteroscedasticity. Finally, separate regression analyses were performed for men and women, resulting in distinct muscle age functions for each sex. The statistical analyses were conducted using R (version 4.3.0; R Foundation for Statistical Computing).

### Ethical Considerations

This study was approved by the Asan Medical Center institutional review board (approval code IRB number 2022-1049; approval date August 4, 2022). The requirement for patient consent was waived.

## Results

### Participants

A total of 537 consecutive patients (mean age 46.9, SD 13.2 y; 345 males and 192 females) were recruited for the study. During the recruitment period, no participants were excluded. Most patients underwent abdominal CT scans for initial evaluation or follow-up of incidental lesions detected on routine abdominal ultrasonography such as pancreatic cysts, gallbladder polyps, and focal lesions in the liver, kidney, or spleen. Some patients underwent abdominal CT scans due to increased levels of blood tumor markers. The demographic characteristics of the participants, including height, weight, and BMI, are listed in Table 1.

**Table 1.** Demographic characteristics of the study participants by age groups.

Characteristics		Age groups (years)					
		20‐29	30‐39	40‐49	50‐59	60‐69	70+
Sex, n							
M^a^		20	98	109	68	35	15
F^b^		20	34	42	51	30	15
Height (cm), mean (SD)							
M		174.6 (5.6)	174.3 (5.3)	174.9 (6.3)	171 (4.4)	169.6 (5.5)	165.5 (6.2)
F		160.4 (5.1)	162.9 (5.8)	161.3 (5.5)	159.1 (4.9)	154.4 (5.1)	154.5 (4.5)
Weight (kg), mean (SD)							
M		80.2 (17.3)	81.6 (14.2)	80.7 (11.9)	74.7 (9.6)	74.6 (8.1)	72.2 (9.5)
F		58.8 (10.4)	59.4 (9.1)	59.6 (8.7)	60 (8.2)	58 (7.3)	59.5 (9.6)
BMI (kg/m^2^), mean (SD)							
M		26.2 (5)	26.8 (3.9)	26.3 (3.1)	25.5 (2.8)	26 (2.7)	26.3 (2.6)
F		23 (4.5)	22.4 (3.2)	22.9 (3.2)	23.8 (3.2)	24.3 (3.2)	24.2 (3.9)

a M: male.

b F: female.

### Fully Automated AI-Integrated Opportunistic CT Screening System

**Figure 3.**
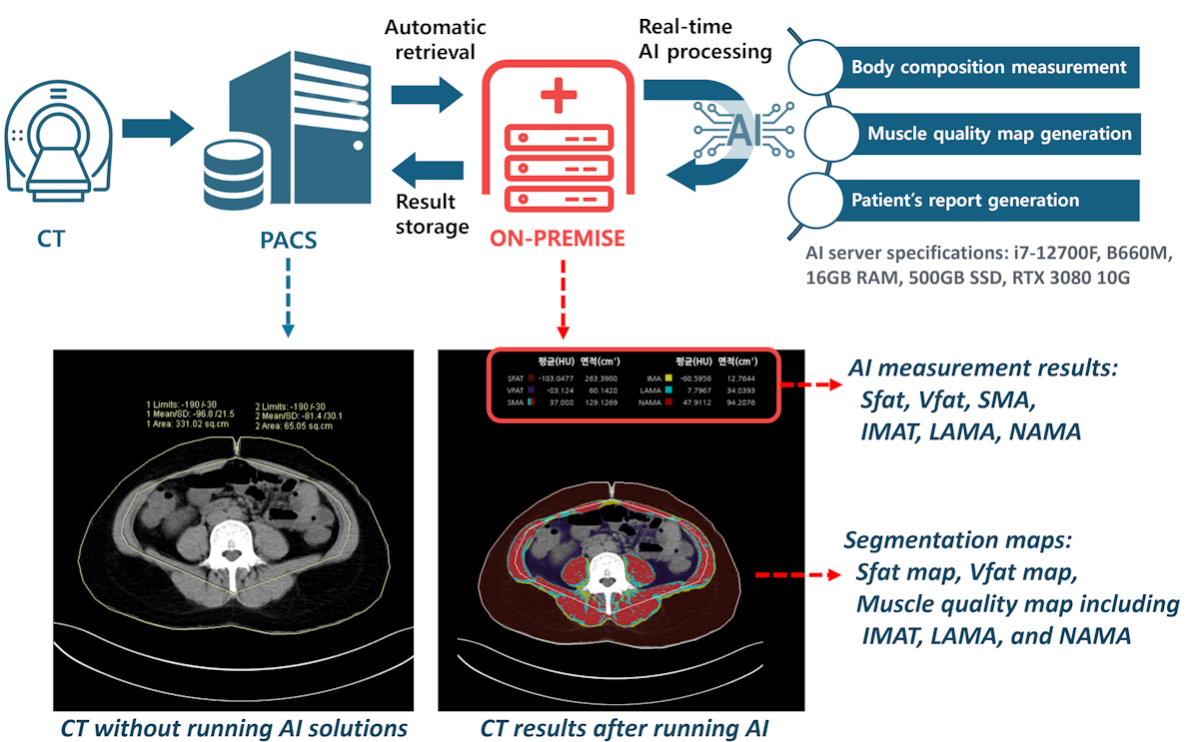
Processes of fully automated AI-integrated opportunistic CT screening system. AI: artificial intelligence; CT: computed tomography; IMAT: inter/intramuscular adipose tissue; LAMA: low-attenuation muscle area; NAMA: normal-attenuation muscle area; PACS: Picture Archiving and Communication System; SFAT: subcutaneous fat area; SMA: skeletal muscle area; VFAT: visceral fat area.

The AI algorithm model was implemented on on-premise servers which were successfully integrated into the PACS in 3 institutions. As illustrated in Figure 3, to establish fully automated AI-integrated opportunistic CT screening system, the following processes were established:

1. Automatic retrieval: the system automatically retrieved anonymized CT images from the PACS without requiring manual intervention. Height and weight information was also extracted and integrated into the Digital Imaging and Communications in Medicine (DICOM) metadata.
2. Real-time AI processing: images were processed in real-time to generate detailed body composition metrics, including measurements of skeletal muscle and adipose tissue.
3. Result storage: the processed results were stored back into the PACS in compliance with DICOM standards.

**Figure 4.**
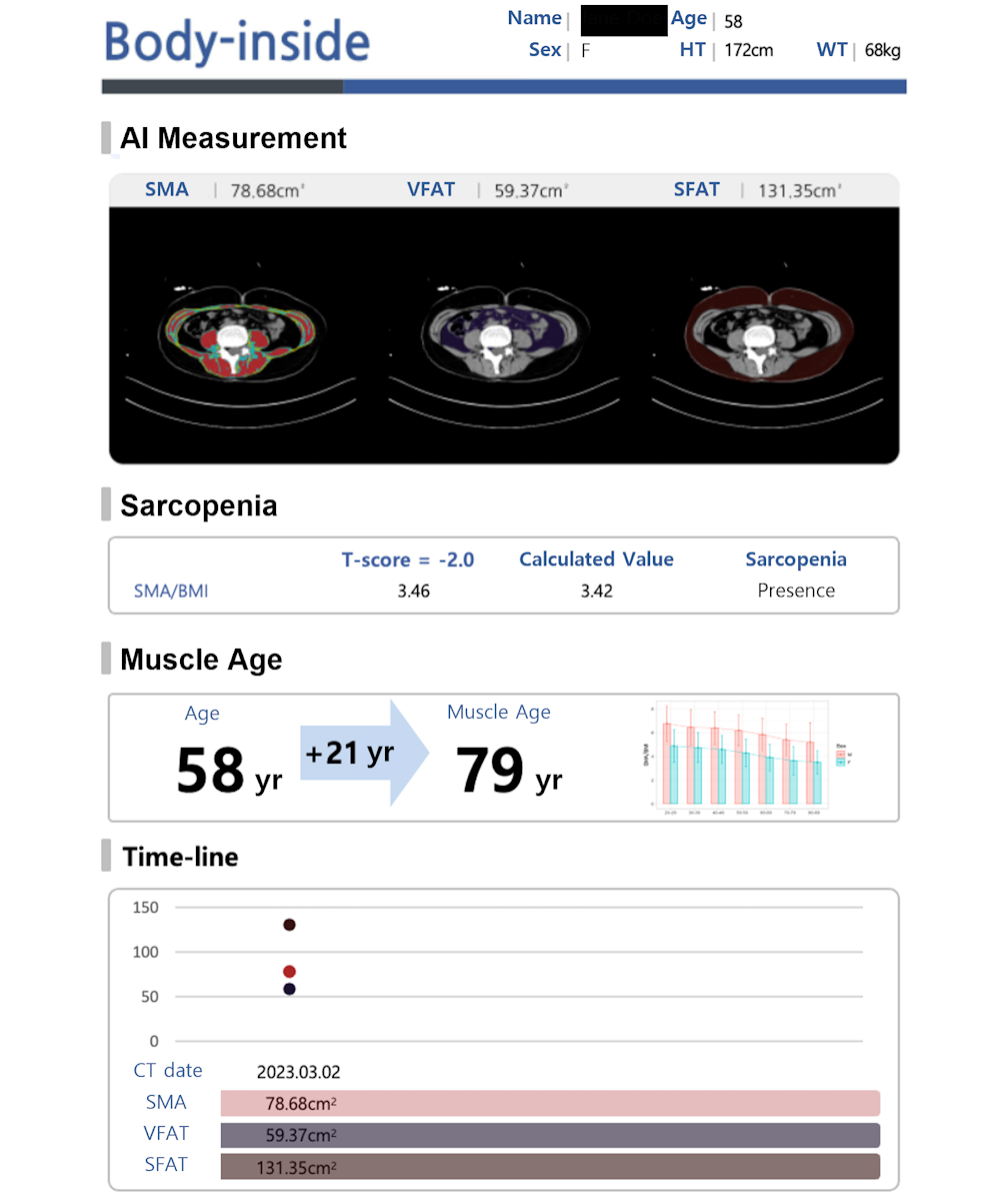
Report form of opportunistic sarcopenia screening results. AI: artificial intelligence; CT: computed tomography; HT: height; SFAT: subcutaneous fat area; SMA: skeletal muscle area; VFAT: visceral fat area; WT: weight.

The fully automated AI-integrated system provided detailed body composition metrics, including muscle quality maps and estimated muscle age, as part of the health check-up reports (Figure 4). The integration of the serial analysis function was added for tracking longitudinal changes. In addition, a muscle age calculator was developed so that clinical users can calculate muscle age manually if DICOM metadata does not contain height and weight information (Video S2 in Multimedia Appendix 2; assessed on the web [15]).

### AI System Performance

The AI system demonstrated robust performance across all evaluated metrics. Among the 537 abdominal CT scans analyzed, the technical success rate was 100%, with no cases requiring manual adjustment or failing due to technical errors.

The mean overall processing time from the completion of CT scan acquisition to the generation of AI results was 4.12 (SD 0.15) seconds per scan, confirming the system’s real-time applicability.

Segmentation accuracy of the AI algorithm, as assessed by the DSC, was 0.974 (SD 0.011), indicating excellent agreement with manual radiologist annotations.

### Opportunistic Screening Results

**Figure 5.**
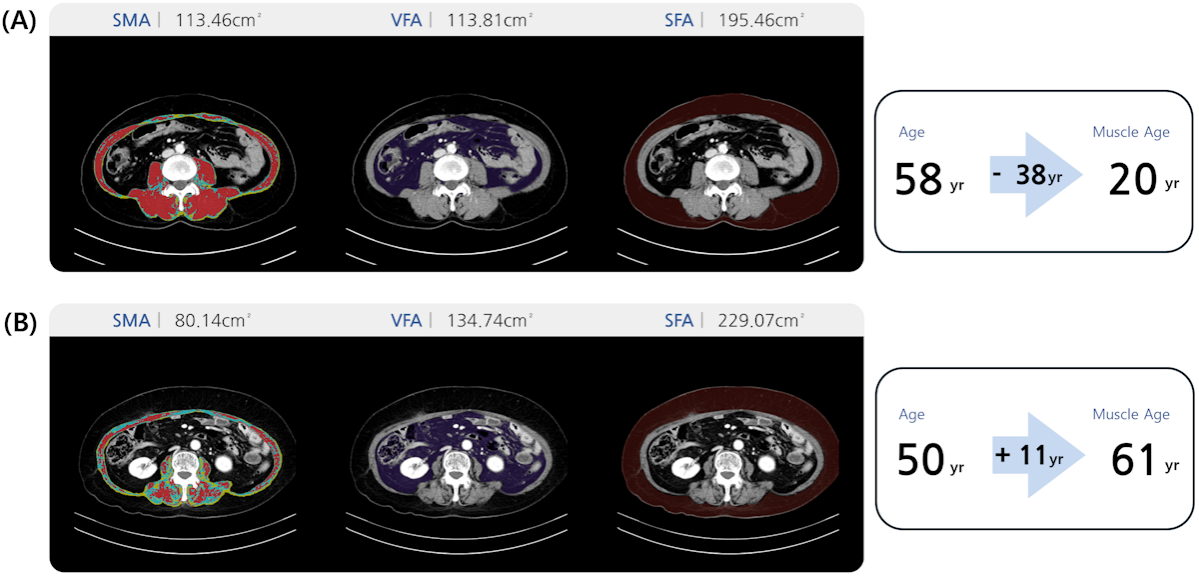
Representative cases with healthy muscles (**A**) and sarcopenia (**B**). (**A**) A 58-year-old female (172 cm, 55 kg) with an SMA/BMI ratio of 6.1, indicating healthy muscle quality, corresponding to the representative SMA/BMI value of a 20-year-old female. (**B**) A 50-year-old female (169 cm, 58 kg) with an SMA/BMI ratio of 4.0, suggesting sarcopenia, corresponding to the representative SMA/BMI value of a 61-year-old female. SFAT: subcutaneous fat area; SMA: skeletal muscle area; VFAT: visceral fat area.

Results of opportunistic screening for body composition are presented in Table 2. In terms of sarcopenia screening, age-related changes in muscle mass and quality were evident in both male and female participants, as NAMA and SMA, indicators of healthy muscle and total muscle, respectively, consistently declined with age. In contrast, LAMA and IMAT, which reflect fat infiltration and reduced muscle quality, showed significant increases across age groups (Figure 5). These trends align with the natural progression of sarcopenia and myosteatosis, emphasizing the impact of aging on muscle health.

**Table 2.** Mean (SD) body composition results in male and female participants across age groups.

	Age groups (years)						
Male	20‐29	30‐39	40‐49	50‐59	60‐69	70+	*P* value^a^
NAMA**^b^**	139.1 (19.8)	132.7 (22.7)	125.7 (20.1)	117.2 (23.2)	106.3 (22)	75.5 (26)	<.001
LAMA**^c^**	28.8 (15.9)	37.4 (17.5)	40.7 (17)	40.3 (15.5)	46.4 (18.2)	59.7 (14.5)	<.001
SMA**^d^**	168 (21.7)	170.1 (25.4)	166.4 (22.9)	157.5 (21.7)	152.6 (18.8)	135.3 (23.2)	<.001
SMA/BMI	6.5 (0.9)	6.4 (0.6)	6.3 (0.7)	6.2 (0.7)	5.9 (0.7)	5.1 (0.6)	<.001
IMA**^e^**	13.3 (7.6)	16.7 (6.9)	16.9 (5.7)	17.2 (5.4)	19 (5.6)	28.4 (7)	<.001
SFAT**^f^**	200.2 (126.8)	220.6 (90.1)	192.3 (59)	158.9 (52.2)	158.4 (47.5)	168.5 (47)	<.001
VFAT**^g^**	92.2 (58)	125.5 (56.6)	152.8 (54.2)	146.5 (56.1)	184 (74.1)	213.9 (73.3)	<.001
Female							
NAMA	81.2 (13.1)	85 (11.7)	73.2 (12.7)	69 (15)	57.4 (14.4)	55.3 (13.9)	<.001
LAMA	23.2 (10.8)	24.8 (6.6)	28.6 (8.6)	33.7 (9.6)	40.1 (8.8)	45.9 (10.9)	<.001
SMA	104.5 (12.2)	110 (11.7)	102.7 (12.2)	102.7 (14.6)	97.6 (11.4)	101.5 (9.5)	<.01
SMA/BMI	4.6 (0.7)	5 (0.7)	4.5 (0.5)	4.3 (0.5)	4.1 (0.6)	4.2 (0.5)	<.001
IMA	10.7 (5.3)	12.4 (4.2)	15.7 (6.6)	17.5 (5.4)	19.9 (5.1)	23.9 (7.1)	<.001
SFAT	192.4 (121.7)	183.1 (70.1)	173.2 (67.9)	180.3 (57.1)	176.2 (60.3)	185.4 (60)	.94
VFAT	45 (23.8)	53.1 (29)	68 (33.7)	89 (41.4)	107 (47.6)	132.5 (61.3)	<.001

a *P* values from 1-way ANOVA.

b NAMA: normal-attenuation muscle area.

c LAMA: low-attenuation muscle area.

d SMA: skeletal muscle area.

e IMA: intramuscular adipose tissue.

f SFAT: subcutaneous fat area.

g VFAT: visceral fat area.

Regarding obesity screening, VFAT increases with age in both genders, highlighting the trend of age-related visceral fat accumulation, a known risk factor for metabolic disorders. SFAT, however, exhibited differing trends between males and females. In males, SFAT decreased in older age groups, while in females, it remained relatively stable across all ages, suggesting a relative preservation of subcutaneous fat. These differences may point to sex-specific mechanisms in fat storage and distribution.

## Discussion

### Principal Results

This prospective multicenter study evaluated the implementation of a fully automated AI-integrated system of body composition assessment for opportunistic CT screening during routine health check-ups. The AI system demonstrated robust performance, with a 100% technical success rate, an average processing time of 4.12 seconds per scan, and high segmentation accuracy (DSC: mean 0.9740.011). The AI system provided comprehensive body composition metrics, including muscle quality maps and estimated muscle age, seamlessly integrated into health check-up reports. These findings validate the reliability, efficiency, and scalability of the AI system for opportunistic sarcopenia screening.

The study revealed significant age-related changes in muscle quality and fat distribution, consistent with the progression of sarcopenia and myosteatosis [11,12,16]. In both males and females, NAMA and SMA declined with advancing age, reflecting a reduction in muscle mass and quality. In contrast, LAMA and IMA, markers of muscle fat infiltration, increased significantly with age. These results underscore the capability of the AI system to detect and quantify meaningful variations in body composition, aligning with physiological expectations and clinical relevance [1].

We used BMI-adjusted skeletal muscle index (SMA/BMI) in our study. Although height-adjusted skeletal muscle index (SMA/height²) is widely used in Western populations, previous research in a large Korean cohort demonstrated that the BMI-adjusted index (SMA/BMI) better reflects age-related muscle decline. Therefore, we adopted SMA/BMI in this study [14].

Notably, our study proposed the concept of muscle age, which provides an individualized estimate of physiological muscle health by projecting the participant’s muscle mass onto the age group with a similar average muscle mass. This metric allows for direct comparison between an individual’s muscle mass and population norms, enabling more nuanced assessment beyond traditional metrics such as chronological age or raw muscle mass, like the concept of biological age [17].

### Comparison With Prior Work

There have been numerous retrospective studies which have assessed AI systems for body composition analysis in terms of segmentation accuracy, technical adequacy, and prognostic utility [11,12,18-20]. Retrospective studies, though valuable, often lack the operational insights necessary to understand the challenges and benefits of integrating AI into real-time clinical workflows. To date, only a few prospective studies have explored the use of AI-based systems in specific clinical contexts, such as routine abdominal CT scans for pancreas cancer follow-up or lung cancer screening programs [21,22].

To the best of our knowledge, this is the first prospective multicenter study to implement a fully automated AI-integrated system for opportunistic screening of body composition in health check-up institutions. By evaluating the system’s performance across diverse institutions, this study bridges the gap between research applications and practical clinical implementation, highlighting the readiness of AI technologies to transition from the research setting to routine health care practice [13]. The successful integration of the AI system into clinical workflows demonstrates its potential use in routine health check-ups by automating time-intensive tasks and report generation. Furthermore, the system’s ability to detect early signs of sarcopenia and myosteatosis offers significant clinical value, as timely interventions can prevent progression to frailty and associated complications [6]. Despite these promising results, integrating AI into institutional PACS presented several challenges, including ensuring seamless data transfer, maintaining compliance with DICOM standards, and obtaining clinical team acceptance. A phased deployment approach and early collaboration between AI engineers, radiologists, and PACS teams were essential to overcoming these barriers. For real-world implementation, we are preparing regulatory approval as a software as a medical device and ensuring compliance with regulations such as the Health Insurance Portability and Accountability Act (HIPAA) and the General Data Protection Regulation (GDPR).

### Limitations

This study has several limitations. First, the study population consisted of participants undergoing routine health check-ups, which may limit the generalizability of the findings to other clinical settings, such as acute care or cancer follow-up. Second, while the AI system demonstrated high segmentation accuracy and efficiency, the study relied on standardized imaging protocols across the participating institutions. Variations in CT acquisition protocols or differences in scanner types at other facilities might affect the system’s performance, necessitating additional validation in diverse imaging environments. Finally, the study focused on technical performance, without incorporating long-term patient outcomes or clinical decision-making for patient care. Despite these limitations, this study represents an important step toward integrating fully automated AI systems into routine clinical practice, offering a foundation for future research to address these gaps.

### Conclusions

This study demonstrated the successful implementation of a fully automated AI-integrated system for body composition assessment using opportunistic CT screening during routine health check-ups, achieving high accuracy, efficiency, and scalability. Significant age-related changes in muscle quality and fat distribution were identified, highlighting the system’s clinical utility in detecting sarcopenia and myosteatosis.

## Supplementary material

10.2196/69940 Multimedia Appendix 1 Video S1: Demo video of the AI solution for automatic L3 level selection and body composition assessment.

10.2196/69940 Multimedia Appendix 2 Video S2: Demo video of the web muscle age calculator, which can be assessed on the web [15].

## Abbreviations

AI: artificial intelligence
CT: computed tomography
DICOM: Digital Imaging and Communications in Medicine
DSC: Dice similarity coefficient
GDPR: General Data Protection Regulation
HIPAA: Health Insurance Portability and Accountability Act
HU: Hounsfield Unit
IMAT: inter- and intramuscular adipose tissue
KAHP: Korea Association of Health Promotion
LAMA: low-attenuation muscle area
NAMA: normal-attenuation muscle area
PACS: Picture Archiving and Communication System
SFAT: subcutaneous fat area
SMA: skeletal muscle area
VFAT: visceral fat area
YOLOv3: You Only Look Once v3
